# Assessing the validity and responsiveness of a generic preference quality of life measure in the context of posttraumatic stress disorder

**DOI:** 10.1007/s11136-023-03432-y

**Published:** 2023-05-14

**Authors:** Sheradyn R. Matthews, Marja Elizabeth, Larissa N. Roberts, Billingsley Kaambwa, Tracey D. Wade, Reginald D. V. Nixon

**Affiliations:** 1 Flinders University Institute for Mental Health and Wellbeing, P.O Box 2100, Adelaide, SA 5001 Australia; 2 College of Education, Psychology and Social Work, Flinders University, P.O. Box 2100, Adelaide, SA 5001 Australia; 3 College of Medicine and Public Health, Flinders University, P.O Box 2100, Adelaide, SA 5001 Australia

**Keywords:** AQoL-8D, PTSD, PCL-5, Responsiveness, Validity

## Abstract

**Purpose:**

There is limited research exploring the usefulness of generic preference-based quality of life (GPQoL) measures used to facilitate economic evaluation in the context of posttraumatic stress disorder (PTSD). The aim of the current study was to explore the validity and responsiveness of a common GPQoL measure (Assessment of Quality of Life 8 Dimension [AQoL-8D]) in relation to a PTSD condition-specific outcome measure (Posttraumatic Stress Disorder Checklist for the DSM-5 [PCL-5]).

**Method:**

This aim was investigated in a sample of individuals (*N* = 147) who received trauma-focused cognitive-behavioural therapies for posttraumatic stress disorder. Convergent validity was investigated using spearman’s correlations, and the level of agreement was investigated using Bland–Altman plots. Responsiveness was investigated by exploring the standardised response means (SRM) from pre-post-treatment across the two measures, which allow the comparison of the magnitude of change between the measures over time.

**Results:**

Correlations between the AQoL-8D (dimensions, utility and summary total scores) and the PCL-5 total score ranged from small to large and agreement between the measures was considered moderate to good. While SRMs were large for the AQoL-8D and PCL-5 total scores, the SRM for the PCL-5 was nearly double that of the AQoL-8D.

**Conclusion:**

Our findings demonstrate that the AQoL-8D has good construct validity but present preliminary evidence that economic evaluations using only GPQoL measures may not fully capture the effectiveness of PTSD treatments.

**Supplementary Information:**

The online version contains supplementary material available at 10.1007/s11136-023-03432-y.

As demand for mental health services increases, effective allocation of health resources to maximise mental health outcomes is paramount [1]. Economic evaluations provide information on the cost-effectiveness of health treatments, therefore providing policymakers with evidence regarding where to strategically invest scarce resources to maximise health gains [2]. Although the use of economic evaluation in mental health conditions such as depression, anxiety and schizophrenia has increased in recent years [3], far less attention has been paid to the cost-effectiveness of posttraumatic stress disorder (PTSD) treatments, a mental health condition that impacts 1.5 million Australian adults annually [4]. Despite recommendations for the inclusion of economic analyses alongside randomised control trials, this rarely occurs and as such, there are substantial gaps in our understanding of the economic impact of particular mental health treatments [3]. Conservative estimates suggest effective and targeted resource allocation in the treatment of PTSD in adult survivors of childhood trauma would save the Australian government 9.1 billion dollars annually [5]. This estimate would inevitably grow if individuals who experienced trauma in their adulthood were included. Effective treatment of PTSD also results in substantial improvements in social functioning and quality of life for individuals [6, 7]. As such, promoting the use of economic evaluations in understudied areas such as PTSD is vital to ensure that there is evidence to guide effective resource allocation.

Cost-effectiveness analysis (CEA) and cost-utility analysis (CUA) are the most commonly used types of economic evaluations [2]. These two methods are identical in how costs are quantified; however, they differ in how health outcomes are measured. In CEAs, outcomes are measured in natural units relevant to the disorder/disease in question (e.g., PTSD symptoms, number of hospital admissions), whereas in CUA the outcome is measured in terms of a generic metric of health [8], most commonly expressed in Quality Adjusted Life Years (QALYs). A QALY represents the quantity and quality of an individual’s life where one QALY is equivalent to one year in perfect health [2]. Generic preference quality of life (GPQoL) instruments (i.e., a measure of health-related quality of life not specific to any particular health condition) or condition-specific preference quality of life (CPQoL) instruments allow the calculation of QALYS. The total scores of these instruments are converted into utility values, which are then multiplied by a specific timeframe to provide the number of QALYS lost or gained over time (see [8] for greater detail regarding how QALYS are derived from GPQoL or CPQoL instruments].

Given that QALYS are a generic measure, they can enable comparison of QALYS gained from different treatments across various condition/disease areas [2]. This contrasts with a CEA approach where comparisons between economic analyses are only possible for studies that use the same condition-specific outcome measure. For this reason, policy bodies such as the Medical Benefits Advisory Committee (MSAC) in Australia and the National Institute for Health and Clinical Excellence (NICE) in the United Kingdom recommend the use of preference-based QoL measures in economic evaluations to facilitate the calculation of QALYS [9, 10]. Policymakers can then make cross-health sector comparisons to better inform decisions regarding the allocation of resources across the entire health sector [2].

There have, however, been inconsistent findings surrounding the usefulness of GPQoL instruments in the field of mental health. A recent review of reviews by Finch et al. [11] found that three common GPQoL instruments—EQ-5D [12], Short Form-6 Dimension (SF-6D) [13], and the Health Utilities Index Mark 3 (HUI-3) [14]—generally performed well in terms of their convergent validity and responsiveness (i.e., a measures ability to detect health change over time) to symptom change following treatment when compared to depression and anxiety measures. However, the EQ-5D, a widely used GPQoL measure in the health and mental health field, performed poorly when compared to specific measures of schizophrenia, bipolar and personality disorders [11, 15]. The authors were unable to comment on the efficacy of the SF-6D and HUI-3 in relation to schizophrenia, bipolar and personality disorder due to the limited studies including these measures. However, individual studies elsewhere have also shown inconsistencies in the validity and responsiveness of these in these conditions [16, 17].

Whilst limited studies have explored this relationship in relation to PTSD, a recent study by Dams et al. [18] compared the EQ-5D to a self-report (University of California Los Angeles PTSD Reaction Index) and a clinician-administered measure of PTSD (Clinician-Administered PTSD Scale for Children and Adolescents [CAPS-CA]) in adolescents and young adults. A moderate correlation was found between the EQ-5D and both PTSD measures (*r*s between − 0.50 and − 0.53). Furthermore, this study found that the EQ-5D’s ability to detect PTSD symptom change after treatment (which was only examined in relation to the CAPS-CA) was weak, either demonstrating non-significant changes over time or associated with small effect sizes between follow-up time points. There is a need for further research and replication to better understand the relationship between GPQoL measures and PTSD symptom measures, particularly in adult populations.

GPQoL instruments may be less useful in certain complex disorders as they typically have limited scope in capturing aspects of mental health. For example, the EQ-5D measures mental health through a single anxiety/depression item [19]. This may explain why GPQoL instruments are more sensitive to changes in anxiety and depression but less so in disorders such as schizophrenia, where core features of the disorder are characterised by other symptoms beyond anxiety and mood problems. Given that mood changes contribute to only one of the four symptom clusters of PTSD (which comprise of reexperiencing, avoidance, negative alterations in mood/cognition, and alterations in arousal), it is possible that common GPQoL measures would also have difficulty capturing changes in the diverse types of PTSD symptoms. Moreover, some GPQoL measures have more than one item measuring mental health (e.g., Short Form-6 Dimension [SF-6D] [13] and Assessment of Quality of Life 8 Dimension [AQoL-8D] [20]), which may better capture more complex mental health disorders. To our knowledge, no studies have examined the validity and responsiveness of GPQoL instruments in relation to assessing change in PTSD symptoms in an adult population, highlighting a need to investigate these relationships.

Accordingly, the current study used pooled data from studies in which individuals had received trauma-focused cognitive-behavioural therapy for PTSD. This study aimed to examine whether a commonly used GPQoL instrument (AQoL-8D) is as valid and responsive as a widely used self-report PTSD symptom measure (the PCL-5). When exploring this relationship, we were interested in examining the construct validity and responsiveness (defined as the ability to measure symptom change in treatment) of our measures. The PCL-5 is one of the field's most commonly used self-report PTSD measures and has consistently demonstrated excellent validity and psychometric properties [21]. The AQoL-8D is designed to provide greater sensitivity to psycho-social health compared to more commonly used GPQoL instruments such as the EQ-5D and HUI3[22].^1^ Over half of the 35 AQoL-8D items combine to form the super dimension ‘psycho-social health’ [20]. As such, this measure may have greater sensitivity to changes in mental health symptoms than other measures. If GPQoL measures adequately capture PTSD symptom change, efforts should be focused on increasing the use of GPQoL measures in PTSD research instead of promoting the use of condition-specific measures to facilitate economic evaluation.

Given that numerous studies have documented that PTSD is associated with poor quality of life [6, 23, 24], it was predicted that the PCL-5 would have a negative correlation with AQoL-8D summary score and utility scores. Moreover, it was predicted that the mental health dimension of the AQoL-8D would have the greatest negative correlation with PCL-5 total scores, compared to the other AQoL-8D dimensions. Given the lack of previous research, no specific predictions were made regarding reliability and responsiveness of the AQoL-8D compared to the PCL-5, thus these analyses constituted a first, exploratory examination of these relationships.

## Method

### Participants

Data were collected from participants who received PTSD treatment at the Flinders University PTSD Clinic, a research-focused intervention clinic, across three different treatment studies*.* The included studies were approved by The Southern Adelaide Clinical Human Research Ethics Committee or the Women’s and Children’s Health Network Research Ethics Committee and informed consent was obtained from all individual participants. To be included in the treatment studies, participants had to be 18 years and older and meet at least 3 of the 4 PTSD symptom clusters, plus all impairment criteria (see *Measures* for details), established using the Clinician-Administered PTSD Scale (CAPS) [25]. Exclusion criteria included severe cognitive impairment, concurrent treatment for PTSD, uncontrolled substance use or psychosis, and individuals that posed imminent harm to themselves or others. Participants completed a battery of questionnaires, including the AQoL-8D and PCL-5 at pre- and post-treatment (2 weeks after ceasing therapy). The final sample included 147 participants (see Table 1 for demographic and trauma-related information).

**Table 1:** Client demographic and trauma information for intent-to-treat sample (*N* = 147)

Characteristics	*M* (SD) or *n* (%)
Age (years)	42.90 (13.07)
Female	100 (68.49)
White ethnicity	120 (82.19)
Index trauma	
Child sexual abuse	27 (18.49)
Adult sexual assault	15 (10.27)
Child physical abuse	8 (5.48)
Motor vehicle accident	14 (9.59)
Witness death	25 (17.12)
Serious injury/threat of death	18 (12.32)
Physical assault	26 (17.80)
Traumatic loss	9 (6.16)
Home invasion/rape	4 (2.74)
Years since index trauma	15.95 (14.48)

Index trauma reflects the trauma for which an individual was seeking treatment

### Treatments

Two of the treatment studies used the same trauma-focused therapy, Cognitive Processing Therapy (CPT) [26], a form of Cognitive-Behavioural Therapy that is a recommended first-line PTSD treatment [27, 28]. CPT involves challenging client’s unhelpful thoughts and behaviours associated with the traumatic event. Various cognitive-behavioural techniques are used, including Socratic questioning, challenging unhelpful beliefs, identifying patterns of problematic thinking, and constructing alternative, more helpful thoughts. Modules in the program specifically focused on how the trauma(s) negatively impacted beliefs about safety, trust, power and control, esteem, and intimacy.

The third treatment study initially used a low-intensity trauma-focused cognitive-behaviour therapy (This Way Up: TWU) [29] from which participants could be stepped up to receive CPT if they did not initially respond to treatment. TWU is a therapist-assisted, guided online self-help approach based on a trauma-focused, cognitive-behavioural protocol. The program involved eight lessons of online material which clients work through each week (see [30] for further details on the treatments provided across the studies). Across all studies the average number of sessions attended was 11.07 (SD = 4.71).

### Measures

*The Posttraumatic Stress Disorder Checklist for the DSM-5* (PCL-5) [31] is a 20-item self-report questionnaire that measures the impact of an individual’s PTSD symptoms over the last month. The PCL-5 captures the four symptom clusters of PTSD as defined by the DSM-5 which include reexperiencing symptoms (cluster B; items 1–5), avoidance symptoms (cluster C; items 6–7), negative alterations in mood/cognition (cluster D; items 8–14) and alterations in arousal (cluster E; items 15–20). Participants rate how bothered they were by a particular symptom on a 4-point scale ranging from 0 (not at all) to 4 (extremely). Scores are combined to create a total severity score ranging from 0 to 80, with greater scores indicating increased PTSD severity. The PCL-5 has demonstrated test–retest reliability of *r* = 0.84, internal consistency of *a* = 0.96 [21] and great discriminant and convergent validity across numerous studies [21, 32]. The internal consistency of the current sample was *a* = 0.83.

*Assessment of Quality of Life 8 Dimension* [20] is a 35-item Generic Preference Quality of Life (GPQoL) instrument that indexes health-related quality of life. The AQoL-8D contains eight dimensions: independent living, relationships, mental health, coping, pain, senses, happiness, and self-worth. Item responses vary from a 4-point scale to a 6-point scale. The scoring algorithm available through https://www.aqol.com.au was used to create a summary score whereby responses are summed, and higher scores indicate greater health-related quality of life. An algorithm is also used to calculate the AQoL-8D utility values applied for economic evaluation. The utility values were determined using a combined Visual Analogue Scale (VAS) and Time Trade-off (TTO) approach based on an Australian general population. AQoL-8D utility values can range from less than 0 (worse than death) to 0 (death) to 1 (good health) [22]. The AQoL-8D has demonstrated internal consistency *a* = 0.96 and test–retest reliability of ICC = 0.91 [20]. The internal consistency of the current sample was *a* = 0.92.

### Statistical analyses

Data were pooled across the three treatment studies. Data were analysed using SPSS (v.28) and Stata (v.17). Descriptive statistics were estimated and the Shapiro-Francia test was used to test the distribution of the PCL and AQoL-8D summary and utility scores, and PCL-5 total scores. Where scores were not normally distributed, non-parametric tests were applied (e.g., Spearman’s correlations). Pre to post-treatment effect sizes were calculated as per Morris [33] and interpreted as follows: < 0.2 = small, 0.5 = moderate, and 0.8 = large [34]. Convergent validity was explored using Spearman’s correlations which were interpreted as per Kaambwa et al. [35]: *r* > 0.30 = weak, 0.40 to 0.50 = moderate, and above 0.50 = strong. The levels of agreement between the instruments were estimated through Bland–Altman plots; these plot the difference between the two instruments against the mean value for each individual person. To construct the plot, the PCL-5 and AQoL-8D utility scores were converted to z scores as the instruments have varying rating scales leading to differences in the magnitude of scores [36, 37]. Before calculating Z scores, instrument totals were power transformed to follow a normal distribution. There were no available clinical or other to determine a priori acceptable limits of agreement in the plots. Given that the mean differences were normally distributed due to the use of z scores, we used the convention that lack of agreement would be suggested if less than 95% of the differences lay outside the limits of agreement, i.e., outside the ‘mean difference ± 1.96 × standard deviation of the differences’ [38, 39]. Responsiveness was measured through comparing the magnitude of change, indexed by the standardised response mean statistic (SRM), between the PCL-5 and AQoL-8D summary and utility scores from pre- to post-treatment. The SRM was calculated as the difference in scores from pre-post-treatment divided by the standard deviation of the difference. SRM values were interpreted as follows: < 0.2 = small, 0.5 = moderate, and 0.8 = large [34]. To account for missing data at post-treatment (28.76% for PCL-5 and 28.08% for AQoL-8D), a linear mixed model approach using restricted information maximum likelihood estimation was adopted to derive the descriptive statistics necessary to calculate the SRM (and this analysis was used to report on treatment outcomes). As the linear mixed model output provides only the standard error, this was used to derive the standard deviation using the following calculation, (SE*$\surd (N))$, in order to calculate the SRM.

## Results

Pre- and post-treatment PCL-5 and AQoL-8D scores can be seen in Table 2. Overall, PTSD treatment was effective, with clients experiencing a significant reduction in PCL-5 scores from pre-post-treatment,* F*(16, 92.85) = 52.99, *p* < 0.001, *d* = 1.70 [CI 1.38, 2.00]. Client’s AQoL-8D summary scores were significantly higher from pre-post-treatment, indicating an overall increase in health-related quality of life *F*(1, 108.01) = 190.87, *p* < 0.001, *d* = 1.35 [CI: 1.10, 1.60]. Similarly, AQoL-8D utility scores also increased from pre-post-treatment, *F*(1, 104.29) = 180.66, *p* < 0.001, *d* = 1.38 [CI 1.11, 1.64].

**Table 2:** Means and standard error of pre-treatment and post-treatment measures

Variable	Pre-treatment	Post-treatment	
	Mean (SE)	Mean (SE)	
PCL-5: PTSD checklist	48.99 (.95)	11.88 (1.28)	
Assessment of quality of life 8 dimension summary total	55.89 (1.04)	71.35 (1.29)	
Assessment of quality of life 8 dimension utility total	0.43 (0.01)	0.66 (0.02)	

**Fig. 1:**
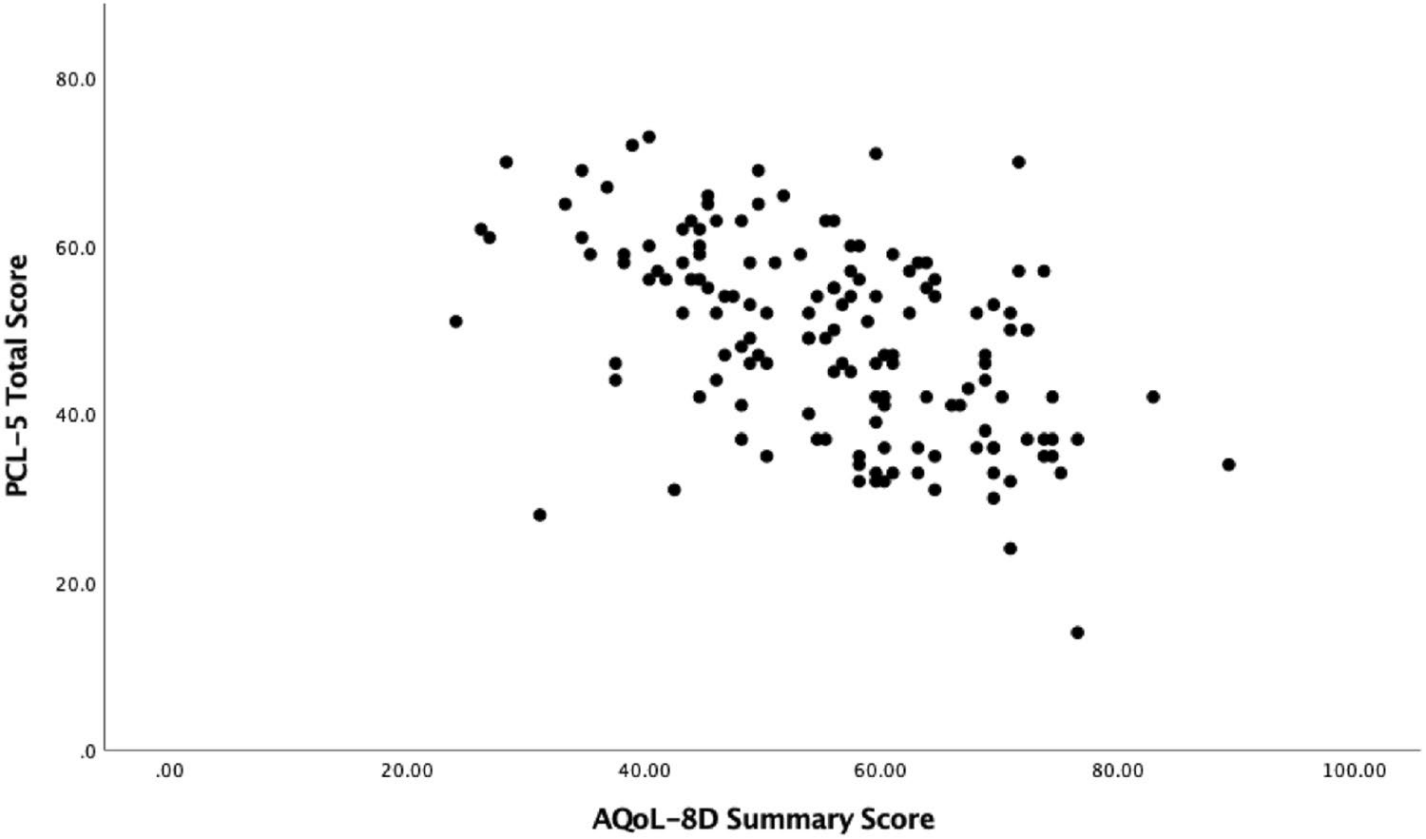
Scatterplot between PCL-5 and AQoL-8D summary scores at pre-treatment

**Fig. 2:**
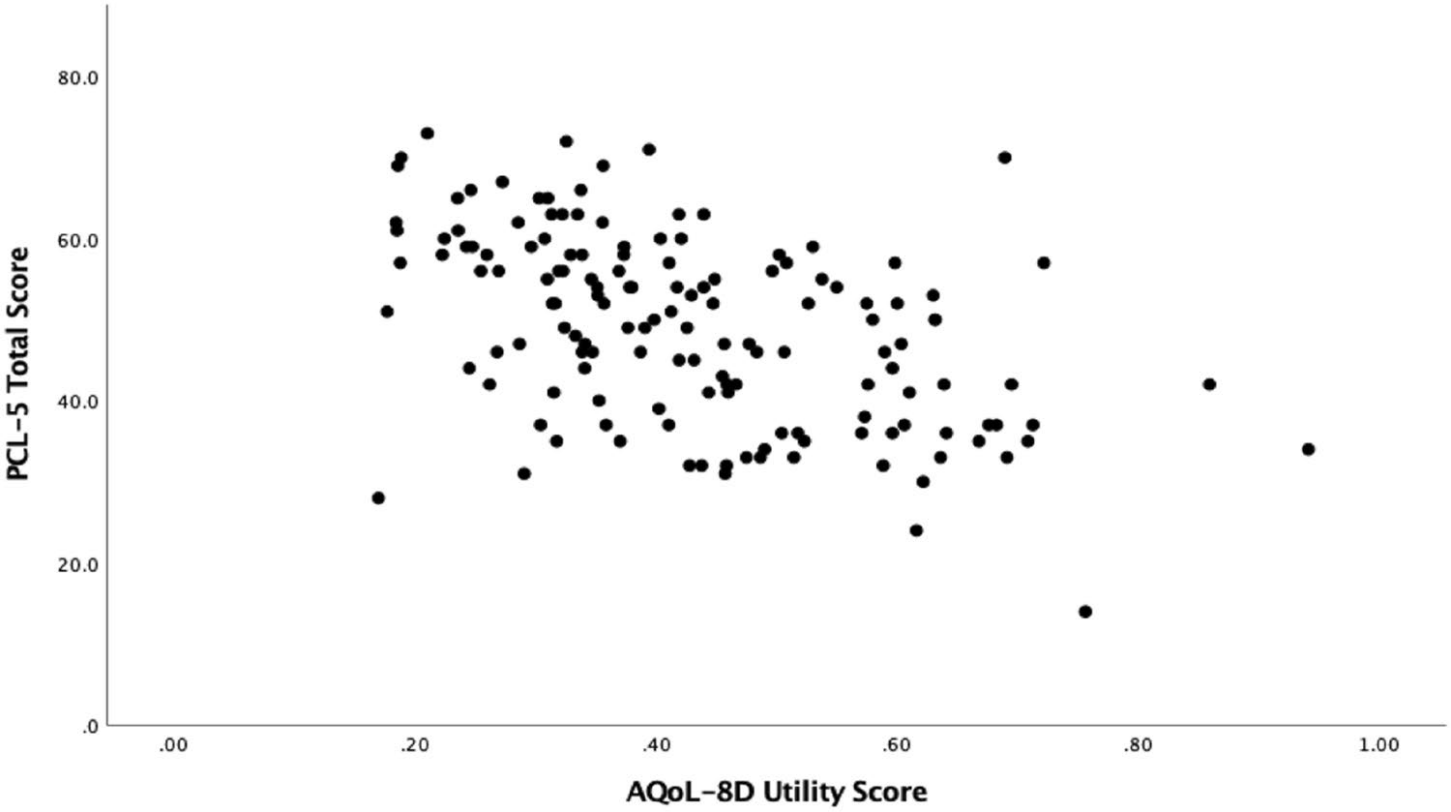
Scatterplot between PCL-5 and AQoL-8D utility scores at pre-treatment

**Fig. 3:**
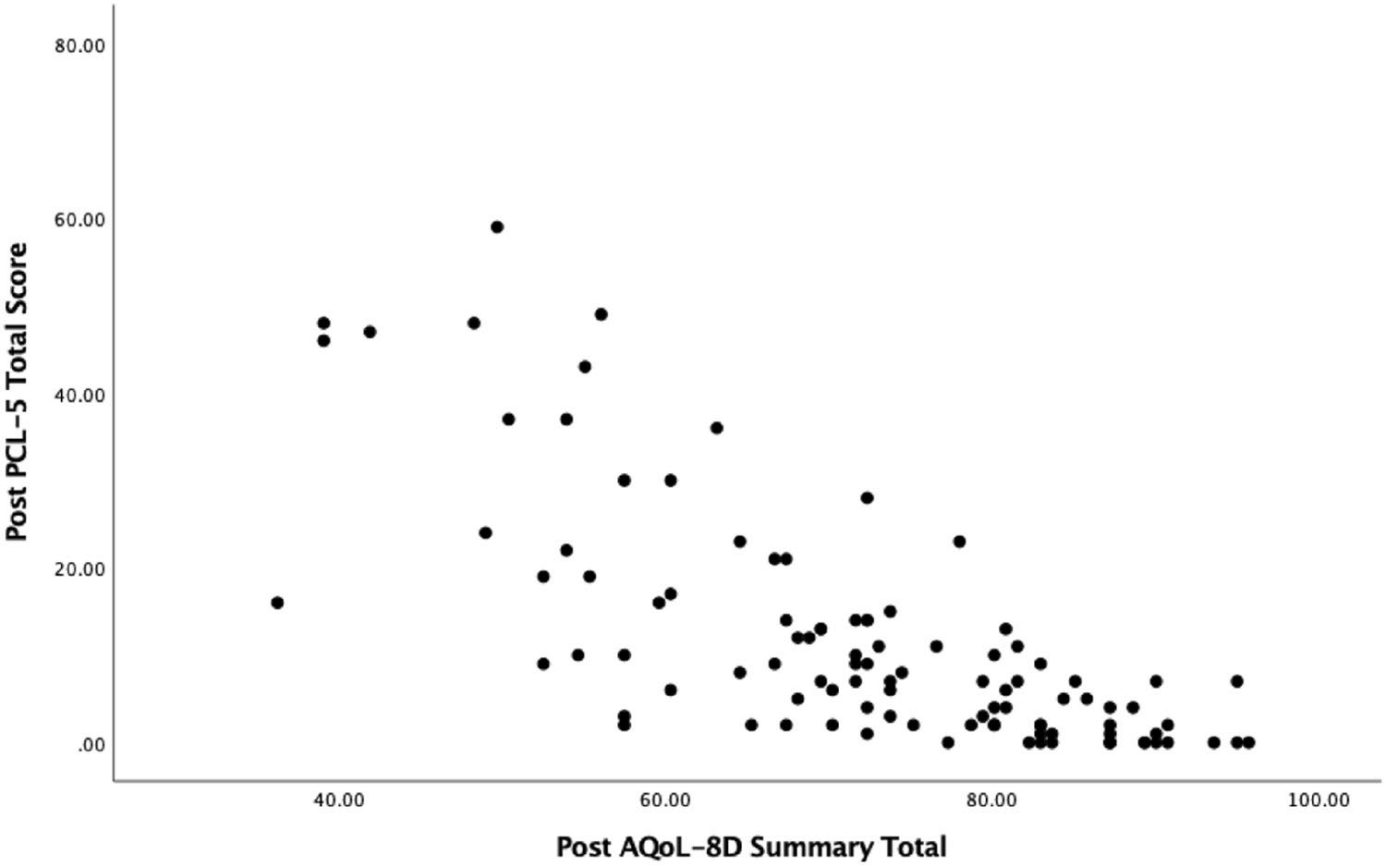
Scatterplot between PCL-5 and AQoL-8D summary scores at post-treatment

**Fig. 4:**
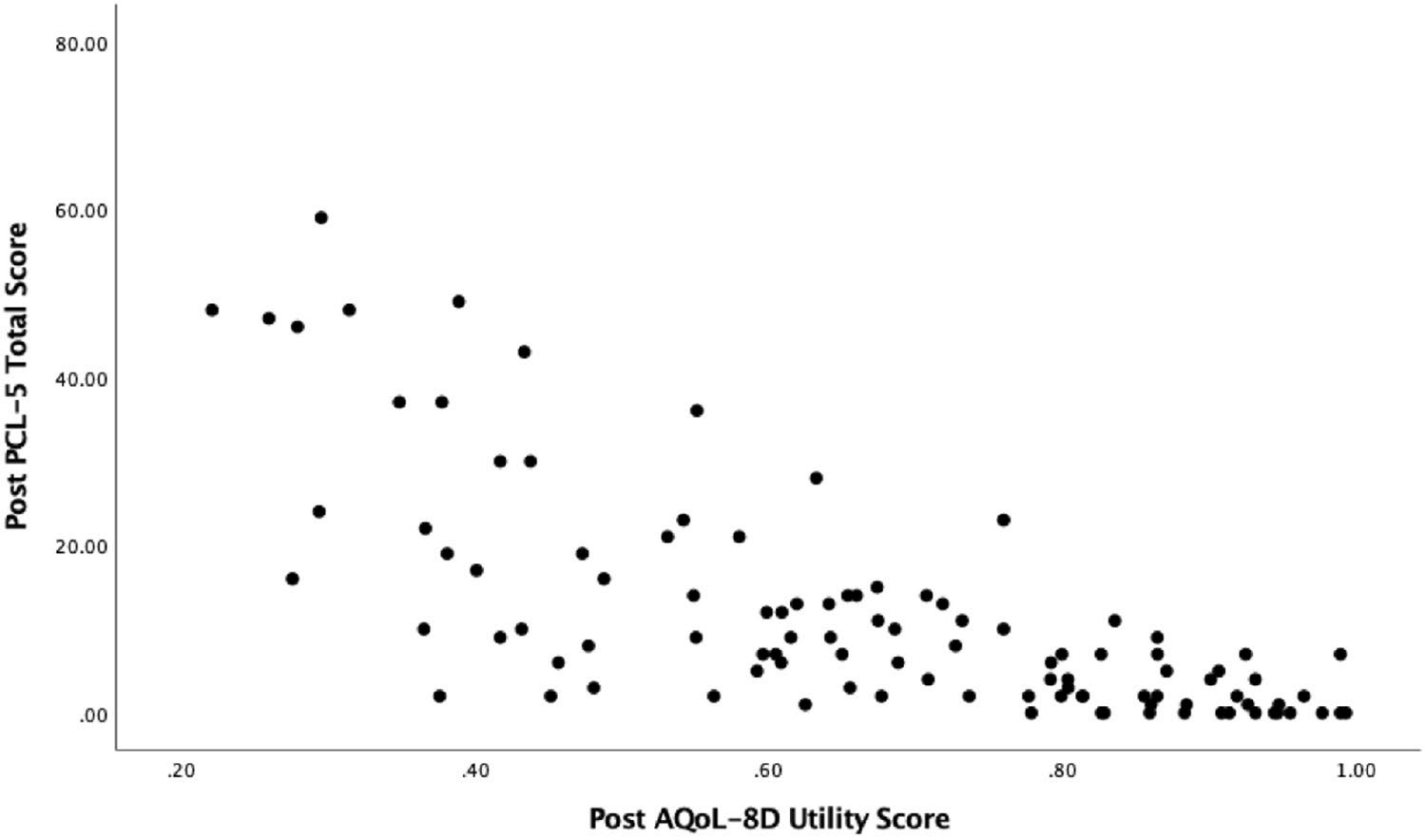
Scatterplot between PCL-5 and AQoL-8D utility scores at post-treatment

To assess convergent validity, Spearman’s correlations were estimated between the PCL-5 symptom clusters and total score and the AQoL-8D dimensions and total scores at pre-treatment and post-treatment (See Table 3). At pre-treatment, the dimensions of independent living, relationships, and the super dimension (physical) had small negative relationships with PCL-5 total scores (*r*’s − 0.24 to − 0.38). AQoL-8D summary total, utility scores and dimensions of mental health, happiness/coping, super dimension (psychosocial) were all moderately correlated with the PCL-5. Pain and senses were the only dimensions not significantly correlated to PCL-5 scores. Similar patterns can be seen when comparing the AQoL dimensions and total scores to post-treatment PCL scores, however, all correlations were larger. Scatterplots showing the relationship between the AQoL-8D summary score and utility score with PCL-5 total scores at pre-treatment and post-treatment can be seen in Figs. 1 and 2 and Figs. 3 and 4, respectively.

**Table 3:** Correlations between pre- and post-treatment PCL-5 symptom clusters and total score, and the AQoL-8D dimension and total scores

	PCL-5				
Variable	Pre PCL-5 total	Reexperiencing	Avoidance	Mood/cognition	Arousal
Pre-treatment scores					
Independent living	− .24*	− .15	− .10	− .26**	− .16
Pain	− .11	− .14	.06	− .10	− .04
Senses	− .09	.001	− .09	− .03	− .14
Mental health	− .53**	−. 35**	− .24**	− .51**	− .37**
Happiness	− .42**	− .17*	− .20*	− .50**	− .24**
Coping	− .48**	− .29**	− .23**	− .48**	− .34**
Relationships	− .38**	− .11	− .19**	− .48**	− .20*
Self-worth	− .41**	− .19*	− .19**	− .48**	− .25**
Super – Physical	− .23*	− .14	− .04	− .19*	− .13
Super – psychosocial	− .52**	− .27**	− .25**	− .60**	− .33**
AQoL-8D summary total	− .52**	− .28**	− .23**	− .56**	− .33**
AQoL− 8D utility total	− .50**	− .29**	− .21*	− .52*	− .34**
Post-treatment scores					
Independent living	− .46**	− .34**	− .26*	− .49**	− .46**
Pain	−.18	− .09	− .09	− .22*	− .20*
Senses	− .37**	− .28**	.37**	− .34**	− .30**
Mental health	− .75**	− .55**	− .56**	− .71**	− .74**
Happiness	− .68**	− .49**	− .50*	− .69**	− .64**
Coping	− .66**	− .47*	− .46**	− .63**	− .70**
Relationships	− .70**	− .48**	− .56**	− .74**	− .61**
Self− worth	− .63**	− .48**	− .43*	− .66**	− .60**
Super – Physical	− .40**	− .27**	− .27**	− .43**	− .37**
Super – psychosocial	− .79**	− .57**	− .60**	− .79**	− .74**
AQoL− 8D summary total	− .75**	− .54**	− .55**	− .72**	− .70**
AQoL− 8D utility total	− .75**	− .55**	− .57**	− .76**	− .70**

*PC*-5 The Posttraumatic Stress Disorder Checklist for the DSM-5, *AQoL*-8*D *Assessment of Quality of Life 8 Dimension. Correlations with moderate-strong effect sizes have been bolded

^*^p < .05; **p < .001

**Fig. 5:**
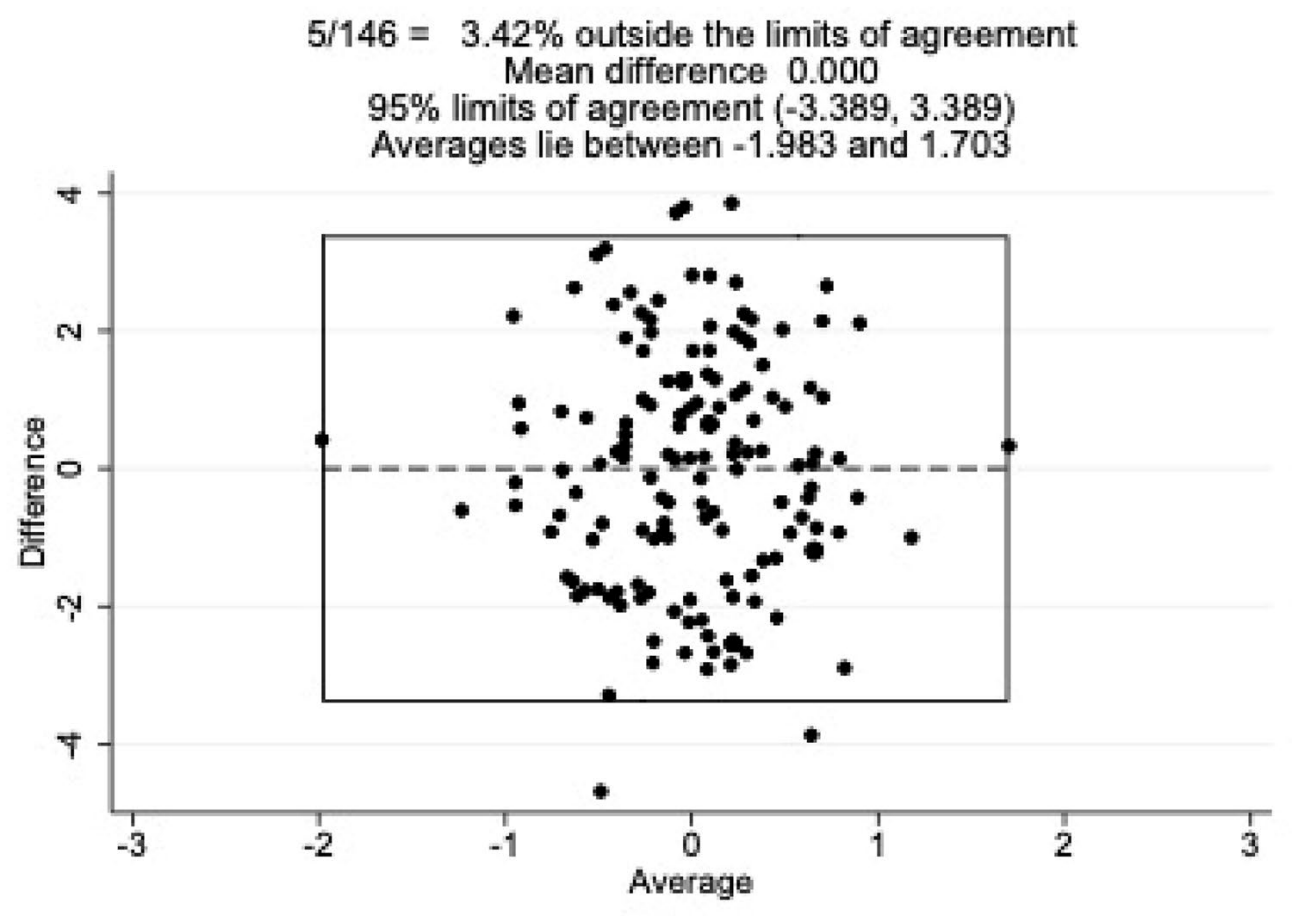
Bland–Altman plots comparing the AQoL-8D utility score and PCL-5 total score

**Fig. 6:**
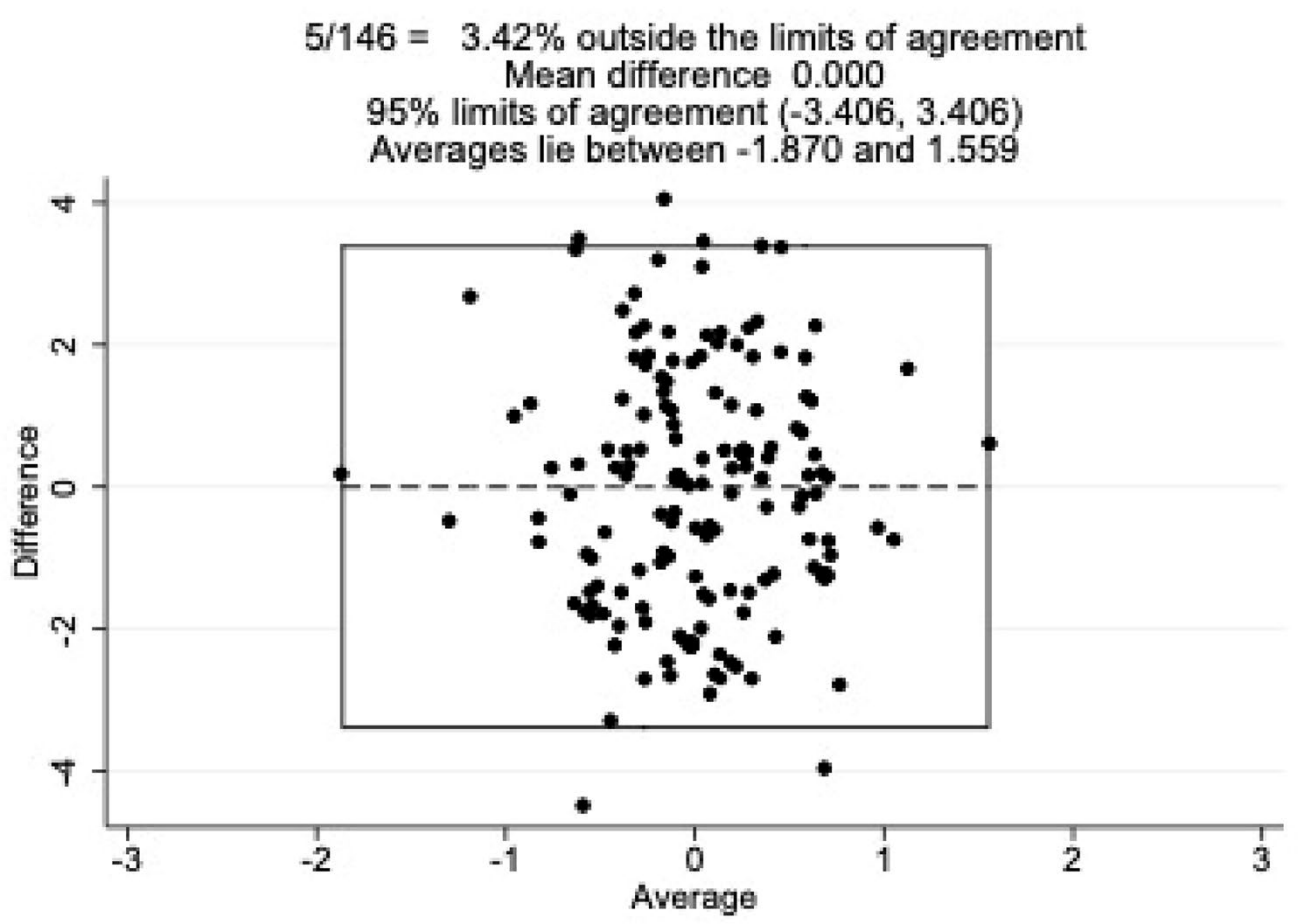
Bland–Altman plots comparing the AQoL-8D summary total score and PCL-5 total score

Agreement between the PCL-5 total and AQoL-8D utility scores was examined using a Bland–Altman plot (see Fig. 5); 3.42% of Z scores fell outside the 95% limits of agreement, suggesting moderate to good agreement between the two measures. The overall limits of agreement were marginal, ranging from -3.39 to 3.39. As the data points appear evenly spread above and below the mean difference of 0, this suggests that there is no consistent bias in the PCL-5 or AQoL-8D compared to the other. A similar pattern of results was found when examining the relationship between the PCL-5 and AQoL-8D summary total (see Fig. 6).

Responsiveness was assessed by comparing the SRM statistic between the AQoL-8D total scores and the PCL-5 total score. As seen in Table 4, clients experienced a large change in PCL-5, AQoL-8D summary and utility scores from pre- to post-treatment. The SRM for the PCL-5 was nearly double that of the AQoL-8D total scores (see table S1 in the online supplementary material for SRM values for each of the AQoL-8D dimensions).

**Table 4:** Standardised response mean (SRM) of client’s pre-post-PTSD treatment change

Variable	Mean change	SD change	SRM
PCL-5 total	37.12	17.56	2.11
AQoL-8D summary score	15.46	13.52	1.14
AQoL-8D utility total	0.23	0.21	1.10

## Discussion

To our knowledge, this study represents the first direct comparison of the validity and responsiveness of a GPQoL instrument and a condition-specific PTSD instrument in an adult population. As expected, there was a negative relationship between client’s PTSD symptoms and their quality of life whereby as PTSD symptoms reduced quality of life increased (correlations ranging from small to large). There was moderate to good agreement between the two measures, as demonstrated by the Bland–Altman plots. Whilst the SRM of both measures was large, the magnitude of change of the PCL-5 was nearly double that of the AQoL-8D summary total and utility score, suggesting that the AQoL-8D was not as sensitive as the PCL-5 to PTSD symptom change over time.

Whilst causality cannot be established from our research, the negative relationship between PTSD symptoms and quality of life is in line with literature demonstrating the pervasive deleterious impact that PTSD can have on an individual’s quality of life [6, 23, 24]. When examining the relationship between quality of life and PTSD based on AQoL-8D dimensions, it was found that the strength of the relationships ranged from small to large. Whilst it was predicted that the mental health AQoL-8D dimension would have the strongest relationship with the PCL-5, it was found that the coping dimension and psycho-social super dimension (including dimensions of mental health, relationships, coping, self-worth, happiness) also shared similar strength relationships. This finding is unsurprising given that increased PTSD severity and mental health difficulties are associated with and can impact one’s ability to cope, overall happiness, self-worth, and relationships [23, 40].

From a health economic point of view, the mean utility total is the most important value derived from the AQoL-8D for the purpose of health economic evaluation, with previous studies suggesting that a moderate correlation may be sufficient to deem a GPQoL measure interchangeable with a symptom-specific measure [41–43]. Therefore, given that the overall AQoL-8D utility total had a strong relationship with the PCL-5 total and that there was moderate to good agreement between the measures, with Z scores showing that the normalised mean scores were all within one standard deviation of each other, our findings show that the AQoL-8D shows some validity in capturing PTSD symptoms. The strength of this relationship is consistent with Dams et al.’s [18] findings which examined the EQ-5D index (or utility total) against both self-report and clinician-administered measures of PTSD. Despite Dams et al. [18] examining different GPQoL and PTSD symptom measures, these consistent findings suggest that there may not be a notable difference in the EQ-5D and AQoL-8D’s ability to capture PTSD severity. However, there is need for replication with a larger and more diverse sample to establish whether there is benefit of one measure of the other.

The AQoL-8D was not as sensitive to change that appeared to occur between pre- and post-treatment as the PCL-5. Whilst the magnitude of both changes was large, change measured on the PCL-5 was nearly double that of the AQoL-8D. This suggests that the AQoL-8D might not fully capture PTSD symptom change over time. This finding is important to consider in the context of economic evaluation. If the aim of the economic evaluation is to determine the quality of an intervention in terms of its ability to reduce PTSD symptoms, using only a GPQoL measure may not adequately index the intervention's effectiveness. Further, there are mixed findings relating to the responsiveness of GPQoLs in other fields of mental health [11], therefore comparing the cost-effectiveness of treatments across disorders (one of the key benefits of using GPQoL measures) may not be a fair comparison if treatment effectiveness is better captured in one disorder compared to the other [17]. Whilst CUA’s are favoured by policymakers for the reasons outlined above, CEA’s are considered acceptable if deemed more appropriate [35]. Whilst our results are preliminary, they do bring into question the responsiveness of the AQoL-8D in relation to PTSD. Accordingly, providing a CEA alongside a CUA in future research would provide a more thorough and accurate depiction of the cost-effectiveness of PTSD interventions.

Our results regarding the responsiveness of the AQoL-8D must be interpreted cautiously—whilst a strength of the study was that responsiveness was evaluated, the secondary data analysis did not allow for more sophisticated analytic approaches. That said, there is no optimum method for measuring responsiveness. However, it is recommended that a distribution-based approach (e.g., examining SRMs over time) be used in conjunction with an anchor-based approach (e.g., use of an external indicator of change to categorise participants into various levels of deterioration or improvement) [35]. Given that our study did not include a measure that could be used as an external indicator of improvement, only distribution estimates could be calculated from pre- to post-treatment. Future work would benefit from conducting estimate and anchor-based analyses when exploring responsiveness, allowing for more robust conclusions to be drawn.

There are additional limitations that should be acknowledged. First, potentially important differences in PTSD symptoms that might be seen in community samples (e.g., gender differences) [44] are not always apparent in treatment-seeking individuals. Future research with both clinical and non-clinical samples would provide more nuanced findings pertaining to the discriminant validity of the AQoL-8D. Second, although we have attributed changes in PTSD symptoms and quality of life to the treatment itself, given that no control group was used, we cannot rule out that other factors may have led to the improvements seen. However, we can feel relatively confident regarding the impact of treatment as it is well established that CPT leads to greater treatment gains compared to non-active control conditions (e.g., those on a waitlist) [45]. Furthermore, there are three other AQoL instruments that are briefer than the AQoL-8D (i.e., the AQoL-4D, AQoL-6D, AQoL-7D). Although these versions capture a smaller number of health dimensions, they have lower participant burden and therefore may be preferred in some settings. Therefore, future research should also consider these practical implications when comparing various GPQoL instruments. Last, although our sample was adequately powered to detect the observed changes (e.g., pre-post-treatment change in PCL-5 and AQoL-8D scores), future research with larger samples might detect even smaller effects. Similarly, a larger sample of PTSD sufferers would enable additional analysis of construct validity of the AQoL-8D (e.g., through confirmatory factor analysis).

Despite these limitations, to our knowledge, this is the first study to make a direct comparison between a GPQoL measure and PTSD symptom measure in an adult population. Whilst there is a need for more research in this area our findings provide preliminary support for the construct validity of the AQoL-8D in individuals with PTSD. To fully capture nuances in PTSD symptom change, studies should conduct cost-effectiveness analyses alongside cost-utility analyses to provide a more accurate depiction of the cost-effectiveness of PTSD treatments.

## Supplementary Information

Below is the link to the electronic supplementary material.

Supplementary file1 (DOCX 17 KB)

## Footnotes

1 Although the AQoL-8D was included in the pooled studies for health economic analyses, the primary aim of these studies was to index clinical outcomes following a psychological intervention. Therefore, the present study represents a secondary analysis of this data.
