# Supplementary material for: Assessing the validity and responsiveness of a generic preference quality of life measure in the context of posttraumatic stress disorder
Source: Qual Life Res. 2023 May 14;32(10):2817–27. doi: 10.1007/s11136-023-03432-y (PMC10474197; doi:10.1007/s11136-023-03432-y)
Supplement: Supplementary file 1 — Supplementary file1 (DOCX 17 KB) [file 11136_2023_3432_MOESM1_ESM.docx]

**Online Supplementary Material**

Matthews, S. R., Elizabeth, M., Roberts, L. R., Billingsley, K., Wade, T. D., & Nixon, R.D.V.

Assessing the Validity and Responsiveness of a Generic Preference Quality of Life Measure in the Context of Posttraumatic Stress Disorder.

*Quality of Life Research.*

Corresponding authors *e-mail address:* [reg.nixon@flinders.edu.au](mailto:reg.nixon@flinders.edu.au) (R.D.V. Nixon) and [sheradyn.matthews@flinders.edu.au](mailto:sheradyn.matthews@flinders.edu.au) (S.R. Matthews).

**Table S1**

*Standardised response mean (SRM) of client’s pre-post PTSD treatment change.*

| Variable | Mean change | | SD change | SRM |
| --- | --- | --- | --- | --- |
| PCL-5 total | 37.12 | 17.56 | | 2.11 |
| AQoL-8D summary score | 15.46 | 13.52 | | 1.14 |
| AQoL-8D utility total | 0.23 | 0.21 | | 1.10 |
| AQoL – independent living | 8.94 | 13.61 | | 0.65 |
| AQoL – Pain | 7.60 | 21.04 | | 0.36 |
| AQoL - Senses | 5.20 | 12.20 | | 0.42 |
| AQoL – Mental Health | 20.87 | 17.99 | | 1.16 |
| AQoL - Happiness | 19.74 | 19.21 | | 1.02 |
| Coping | 20.68 | 20.95 | | 0.99 |
| Relationships | 14.32 | 17.82 | | 0.80 |
| Self-worth | 22.27 | 24.89 | | 0.89 |
| Super – physical | 7.30 | 10.51 | | 0.69 |
| Super – psychosocial | 18.91 | 16.51 | | 1.15 |
